# Towards the Identification of New Biomarkers in Saliva and Serum for Treatment Monitoring of Equine Gastric Ulcer Syndrome: A Liquid Proteomic Approach

**DOI:** 10.3390/ani14213105

**Published:** 2024-10-28

**Authors:** Alberto Muñoz-Prieto, Ivana Rubić, Dina Rešetar Maslov, Juan Carlos González-Sánchez, Vladimir Mrljak, Jose Joaquín Cerón, Sanni Hansen

**Affiliations:** 1 Interdisciplinary Laboratory of Clinical Analysis (Interlab-UMU), Regional Campus of International Excellence ‘Campus Mare Nostrum’, University of Murcia, Campus de Espinardo s/n, 30100 Murcia, Spain; jjceron@um.es; 2 Laboratory of Proteomics, Internal Diseases Clinic, Faculty of Veterinary Medicine, University of Zagreb, 10000 Zagreb, Croatia; irubic@vef.unizg.hr (I.R.); drmaslov@vef.unizg.hr (D.R.M.); vmrljak@vef.hr (V.M.); 3 BioQuant, Heidelberg University, Im Neuenheimer Feld 267, 69120 Heidelberg, Germany; juan-carlos.gonzalez@bioquant.uni-heidelberg.de; 4 Department of Veterinary Clinical Sciences, Section Medicine and Surgery, University of Copenhagen, Agrovej 8, 2630 Taastrup, Denmark; sannih@sund.ku.dk

**Keywords:** EGUS, monitoring, proteome, saliva, serum

## Abstract

Equine gastric ulcer syndrome (EGUS) is a common condition in horses. This study explores the use of liquid proteomics to identify new biomarkers in saliva and serum to monitor EGUS treatment. The proteomes of horses with EGUS before and after a successful treatment with omeprazole were analysed. In saliva, 503 proteins were identified, with 7 upregulated and 6 downregulated post-treatment. Among the proteins that changed, there was an increase in vimentin, linked to wound healing, and a decrease in podocalyxin, associated with tissue damage. In serum, 206 proteins were found, with significant changes in 5. Keratin type I increased, supporting epithelial integrity, whereas immunoglobulin lambda decreased, indicating a reduced immune response. Gene ontology analysis revealed a decrease in immune-related pathways after successful treatment. Overall, 13 proteins in saliva and 5 in serum showed significant changes after treatment, highlighting the differential responses of saliva and serum in EGUS. This report creates new avenues for discovering potential biomarkers to monitor EGUS treatment, which is of high importance for the management of this prevalent disease.

## Simple Summary

Equine gastric ulcer syndrome (EGUS) is a widespread issue in horses. In terms of monitoring EGUS treatment, follow-up gastroscopies are recommended to visualise if the treatment has efficiently resulted in healing progress in the ulcers. The use of analytes measured in non-invasive samples like saliva for treatment monitoring could offer several benefits, such as the welfare of the horse, reduced expenses for the owner, and a more easily obtained sample material for the veterinarian. Moreover, investigating the same analytes in serum could provide valuable comparisons, as well as the serum sample itself being a less invasive option for biomarkers compared to the gastroscopy procedure. The study highlights that saliva and serum show changes in their composition after a successful EGUS treatment when analysed with liquid proteomics. The protein changes in saliva and serum were different, and saliva showed a higher number of proteins that changed compared to serum. Therefore, saliva and serum can be sources of biomarkers to detect the healing of gastric ulcers in horses, opening new possibilities for the easier and more effective monitoring of horse health during treatment.

## 1. Introduction

Equine gastric ulcer syndrome (EGUS) is highly prevalent worldwide, being currently considered as one of the most common diseases in horses [1,2]. The high prevalence of this disease is probably due to the intensive management and high performance requirements of equine species [3]. EGUS comprises two different entities: equine squamous gastric disease (ESGD) and equine glandular gastric disease (EGGD), which can be diagnosed individually or together. It has been described that the causes and mechanisms leading to each disease are different [4]. ESGD arises when the squamous mucosa of the stomach is compromised, usually after excessive exposure to acids. Factors that can induce ESGD include diets high in sugar and starch [5], which lead to a higher production of volatile fatty acids in the stomach. This results in a decrease in the pH of the upper layer of the gastric fluid. In addition, a lack of long-stemmed roughage in the diet due to the use of diets that are too refined and require less chewing can reduce saliva production, leading to insufficient buffering and heightened acid exposure [2,4,6,7,8]. Factors that are linked to EGGD pathogenesis include stressful situations and high doses or the long-term administration of nonsteroidal anti-inflammatory drugs [9]. In addition, EGGD is associated with immune activation and inflammation of the glandular mucosae [10].

Saliva is increasingly being used for biomarker analysis related to animal health and welfare. It offers several advantages over blood samples, including being non-invasive, easy to collect, and not causing the stress associated with venipuncture [11]. EGUS is associated with changes in various saliva analytes related to various physiological mechanisms such as immune status and inflammation [like adenosine deaminase (ADA) [12] or redox status [like the ferric reduction ability of saliva (FRAS), uric acid, and advanced oxidation protein products (AOPPs)] [13,14]. Some of these analytes, such as uric acid, triglycerides, and calcium, can potentially differentiate horses with EGUS from horses with other diseases.

Very few ulcers heal by themselves if no management changes are made and the horse is kept in training [15,16]. Omeprazole, a proton pump inhibitor, is a drug used for EGUS treatment, being more effective in cases of ESGD [17]. EGGD, on the other hand, has a low treatment success rate and a high recurrence rate, which makes this syndrome a treatment challenge [18,19,20]. In terms of monitoring EGUS treatment, follow-up gastroscopies are recommended to visualise if the treatment has efficiently resulted in healing progress in the ulcers [21]. The use of analytes measured in non-invasive samples like saliva for treatment monitoring could offer several benefits, such as the welfare of the horse, limited expenses for the owner, and more easily obtained sample material for the veterinarian. Moreover, investigating the same analytes in serum could provide valuable comparisons, as well as the serum sample itself being a less invasive option for biomarkers compared to the gastroscopy procedure, which includes 16 h of fasting, sedation, and the gastroscopic procedure itself.

Two previous reports have studied the possible changes in salivary analytes when monitoring EGUS treatment. In one, in-gel proteomics was used to identify changes in saliva biomarkers with EGUS treatment. This paper identified proteins related to the immune system, the defensive environment of the stomach, and inflammation that changed after treatment [12]. Additionally, a report on treatment monitoring, in which 17 analytes integrating a biochemical panel were evaluated in saliva, showed that bicarbonate, urea, adenosine deaminase (ADA), and creatine kinase (CK) changed after successful treatment [22]. In addition to these two previous approaches (gel proteomics and a general biochemical panel), liquid proteomics, a more sensitive technique, could detect and provide further information on changes in analytes of interest.

The hypothesis of this work is that there are changes in saliva and serum proteomes of horses with EGUS when treated successfully, that could be detected by liquid proteomics. Therefore, this study aimed to evaluate the possible proteomic changes in saliva and serum in horses at the time of EGUS diagnosis and after successful treatment.

## 2. Materials and Methods

### 2.1. Animals

A total of ten horses (seven geldings and three mares) were included in the study. The sample materials from horses applied in this study were obtained from the Large Animal Teaching Hospital at the University of Copenhagen from August 2020 to August 2023. Each horse underwent a thorough examination, which included evaluations of behavioural changes, medical history, and clinical parameters [such as weight, body condition score (BCS) on a nine-point scale previously published by Henneke et al. [23], heart rate, respiratory rate, rectal temperature, mucous membrane colour, capillary refill time, and intestinal sounds]. Additionally, haematological and biochemical tests were performed.

Following the methodology previously described [24], all horses underwent gastroscopy after a 16 h fasting period. The images obtained from the gastroscopy were used to diagnose ESGD and EGGD according to the ECEIM Consensus Statement guidelines [21,25]. ESGD was diagnosed if a horse scored above 1 on the 0–4 ESGD grading scale, while EGGD was diagnosed with a score above 1 on the 0–4 EGGD grading scale [26].

Horses were eligible for inclusion in the study if they (1) were diagnosed with both ESGD and EGGD and had no other health conditions, including other gastrointestinal disorders, and (2) underwent successful treatment. After confirmation of the EGUS diagnosis by gastroscopy, all included horses were treated with oral omeprazole at 4 mg/kg (gastrogard, Boehringer Ingenheim, Denmark) one hour before feeding in the morning for six weeks. The treatment was deemed successful if the ESGD and EGGD scores were reduced to 0–1.

### 2.2. Paired Serum and Saliva Sampling

Saliva samples were systematically obtained from all horses prior to intravenous sedation (Domosedan vet 10 μg/kg, Orion Corporation, Espoo, Finland) and butorphanol (Dolorex 10 μg/kg, Intervet International B.V, Boxmeer, The Netherlands) and immediately following placement in the examination stock for the gastroscopic procedure (endoscope, length 3 m and diameter 0.8 cm, Kruuse, Langeskov, Denmark). A sponge was utilised for saliva collection, the samples of which were subsequently transferred into Salivette tubes. These tubes were immediately sent to the laboratory within one hour of the collection.

Blood samples were collected immediately after saliva collection through venipuncture of the jugular vein. Upon arrival at the laboratory, the saliva and blood tubes were centrifuged at 3000× *g* for 10 min at 4 °C and stored at −70 °C pending analysis. No serum samples had visual gross haemolysis, and no saliva samples had evidence of blood contamination.

### 2.3. Sample Preparation for Tandem Mass Tag-Labelled Mass Spectrometry

Total protein concentration in serum and saliva was determined using a Merck BCA protein assay kit (MiliporeSigma, Burlington, MA, USA), as per the manufacturer’s protocol. For protein digestion, 35 µg of serum or saliva proteins per sample and an internal standard were adjusted to 50 µL with 0.1 M triethylammonium bicarbonate (TEAB, Thermo Fischer Scientific, Waltham, MA, USA). Bottom-up and multiplex preparation, nano-liquid chromatography with tandem mass spectrometry (nano-LC-MS/MS) analysis, data processing, and statistical analysis were performed as previously described [27]. Briefly, reduction with 200 mM dithiothreitol (Sigma-Aldrich, Merck KGaA, Darmstadt, Germany) at 55 °C for 60 min and alkylation with 375 mM iodoacetamide (Sigma-Aldrich) at room temperature (RT) for 30 min in the dark were followed by overnight acetone precipitation at −20 °C. Protein pellets obtained after centrifugation were dissolved in 50 µL of 0.1 M TEAB. Trypsin Gold (Promega, Madison, WI, USA) was prepared at 1 mg/mL in 0.1 M TEAB and added to the protein solution at a 1:35 ratio for overnight digestion at 37 °C. TMT-6-plex reagents (Thermo Scientific) were prepared according to the manufacturer’s protocol. Labelling proceeded for 60 min at RT and was quenched with 5% (*v*/*v*) hydroxylamine (Sigma-Aldrich). Four TMT 6-plex mixtures were prepared, and aliquots were vacuum-dried for nano-LC–MS/MS analysis.

### 2.4. Nano-LC–MS/MS Analysis and Row Data Processing

High-resolution nano-LC–MS/MS separation and detection of TMT-labelled serum and saliva peptides were performed on an UltiMate 3000 RSLCnano system (Dionex, Germering, Germany) coupled with a Q Exactive Plus Hybrid Quadrupole-Orbitrap mass spectrometer (Thermo Fisher Scientific, Bremen, Germany). Vacuum-dried peptides were dissolved in 0.1% formic acid (VWR, Avantor, Radnor, PA, USA) in 2% acetonitrile (Honeywell International, Charlotte, NC, USA) and ultrapure water (Supelco, Sigma-Aldrich). Peptide trapping, desalting, nano-LC–MS/MS analysis, and Top8 data-dependent acquisition (DDA) in positive-ion mode were performed as described previously [27]. The trap column (C18 PepMap100) and the analytical column (PepMap™ RSLC C18) were both from Thermo Fisher Scientific. Peptides were trapped for 12 min at 15 µL/min. Mobile phase A (0.1% formic acid in water) and mobile phase B (0.1% formic acid in 80% acetonitrile) were used for separation. The gradient increased with mobile phase B from 5% to 45% over 120 min, then to 90% over 2 min; it was held for 2 min and re-equilibrated at 5% for 20 min at 300 nL/min. The mass spectrometer operated in full MS scan mode (*m*/*z* 350.0 to 1800.0) with a resolution of 70,000 and an injection time of 120 ms. The AGC target was 1 × 10^6^ ± 2.0 Da, with a 30 s dynamic exclusion. HCD fragmentation used collision energies of 29% and 35% NCE, with a resolution of 17,500 and an AGC target of 2 × 10^5^. Precursor ions without an assigned charge state and those with a charge state above +7 were excluded from fragmentation.

Raw data processing, protein identification, and relative quantification were conducted using Proteome Discoverer software (v. 2.3, Thermo Fisher Scientific) with the SEQUEST algorithm and *Equus caballus* database. Equus caballus DB from Uniprot/Swissprot was used for protein identification (downloaded on 2 April 2024). The parameters in Proteome Discoverer included two missed trypsin cleavages, a precursor mass tolerance of 10 ppm, a fragment mass tolerance of 0.02 Da, fixed carbamidomethyl (C) modification, and dynamic modifications of oxidation (M), deamidation (N and Q), and TMT 6-plex (K, peptide N-terminus). The Percolator algorithm calculated a false discovery rate (FDR). At least two unique peptides and 1% FDR were required for confident protein identification. Relative protein quantification was achieved by correlating reporter ion intensities from MS/MS spectra with selected peptides, normalising results between six 6-plexes using internal standards, and within one 6-plex using total peptide amounts.

### 2.5. Statistical Analysis

The raw proteomic data were normalised using the median to reduce variability and ensure sample consistency by dividing the spectral profile (intensities of variables) of an object by the median of all the intensities for that object [28]. The data underwent a square root transformation to achieve a more normal distribution. Additionally, Pareto scaling was applied for centring, emphasising the more significant variances while retaining the structure of the smaller ones. For the statistical analysis, features with more than 50% missing data were excluded, while the remaining missing features were imputed using 1/5 of the minimum positive value of each variable. A volcano plot was employed to study the altered proteins in horses with EGUS before and after treatment. A fold change threshold of 2.0 was used for this analysis, with a raw *p*-value cutoff set at 0.05, to identify statistically significant changes in the data. Additionally, the false discovery rate (FDR) was calculated for the proteins that varied significantly after volcano plot analysis, using the Benjamini–Hochberg method. This approach was applied to adjust the *p*-values obtained from the *t*-tests in order to account for multiple comparisons and estimate the rate of false positives.

To functionally characterise the differentially expressed proteins in saliva and serum, gene ontology (GO) enrichment analyses were performed using the Cytoscape plugin ClueGo (version 2.5.10) and its functionalities to fuse and group functionally related terms to reduce redundancy. The ontologies used were updated on 31 August 2024. Proteins and significantly enriched GO terms (*p*-value < 0.05) were represented in functionally grouped networks.

The data of the EGGD and ESGD scores were assessed for normality, showing a non-parametric distribution. Therefore, the statistical significance for comparing the EGGD and ESGD scores was assessed through the paired Wilcoxon matched-pairs signed-rank test. Descriptive data are expressed as median and interquartile range (IQR). Data were considered significant if *p* < 0.05. Descriptive data analysis was performed in the GraphPad Prism software for MAC (version 9.5) (GraphPad Software, Boston, MA, USA).

## 3. Results

### 3.1. Characteristics of the Horses Included in the Study

The horses included in this study presented a median age of 11 (IQR = 9–12.75) years, a median body weight of 552.5 (IQR = 490–573) kg, and a median BCS of 5 (IQR = 5–6). The horses showed a significant decrease in the median ESGD score after treatment (0.5, IQR = 0–1) compared to before treatment (2, IQR = 1–3) (*p* = 0.023) and in the EGGD score after treatment (0, IQR = 0–1)) compared to before treatment (2.5, IQR = 1–3) (*p* = 0.007).

### 3.2. Proteomic Changes in the Saliva and Serum of Horses with Equine Gastric Ulcer Syndrome after Successful Treatment

**Figure 1:**
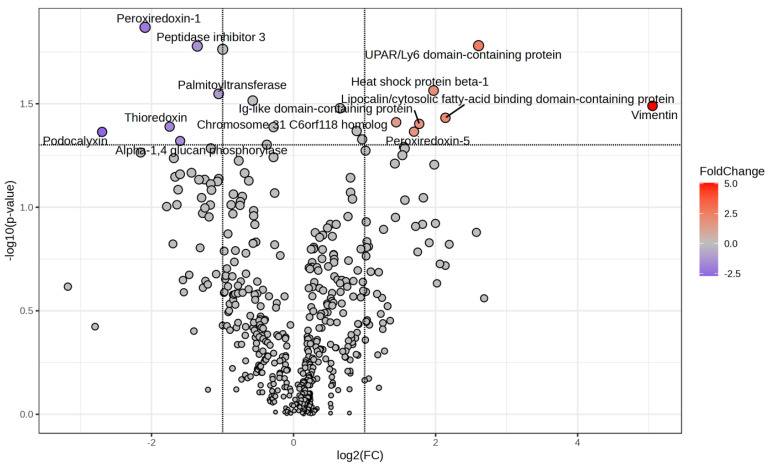
Volcano plot of the salivary proteins showing significant variation (FC threshold = 2; *p* value < 0.05) in horses with equine gastric ulcer syndrome after treatment.

**Figure 2:**
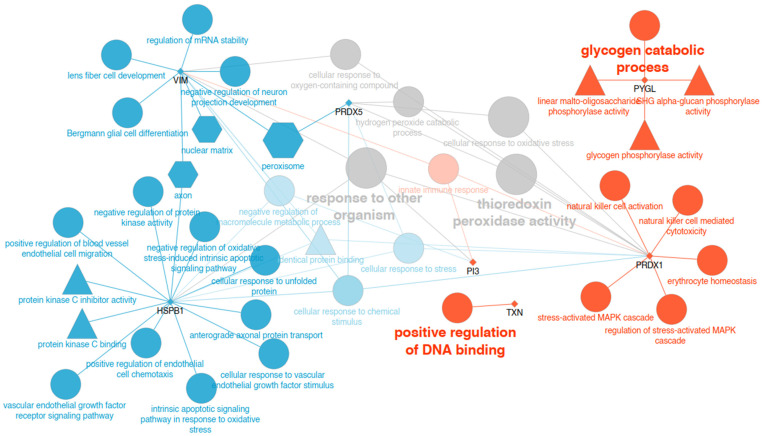
Network-like visualisation of significantly enriched GO annotations of the proteins found differentially expressed in saliva. Small, diamond-shaped nodes with black labels represent proteins. Edges link proteins with their corresponding, significantly enriched GO terms. GO terms are represented with shapes according to the ontology they belong to (‘cellular component’ (hexagons), ‘biological process’ (circles), and ‘molecular function’ (triangles)) and with a size proportional to their significance (the lower the *p*-value, the larger the node size). Over-expressed proteins and their GO terms are in blue, under-expressed proteins and their terms are in red, and grey indicates that a term is equally shared between over- and under-expressed proteins.

A total of 503 proteins were identified in the saliva of horses with EGUS. Among these, seven proteins showed higher and six showed lower abundances after treatment (Table 1). The proteins with the highest abundance were vimentin, UPAR/Ly6 domain-containing protein, and Lipocalin/cytosolic fatty-acid-binding domain-containing protein. Regarding proteins with lower abundance, podocalyxin, peroxiredoxin-1, and thioredoxin showed the highest changes (Figure 1). GO analysis showed that the main upregulated GO terms in saliva were peroxisome (*p* < 0.01), cellular response to chemical stimulus (*p* < 0.01), and cellular response to stress (*p* < 0.01). On the other hand, the main downregulated GO terms were immune innate response (*p* < 0.01), positive regulation of DNA binding (*p* = 0.018), glycogen catabolic process (*p* = 0.018), and natural killer cell activation (*p* = 0.018) (Figure 2, Supplementary Table S1).

**Figure 3:**
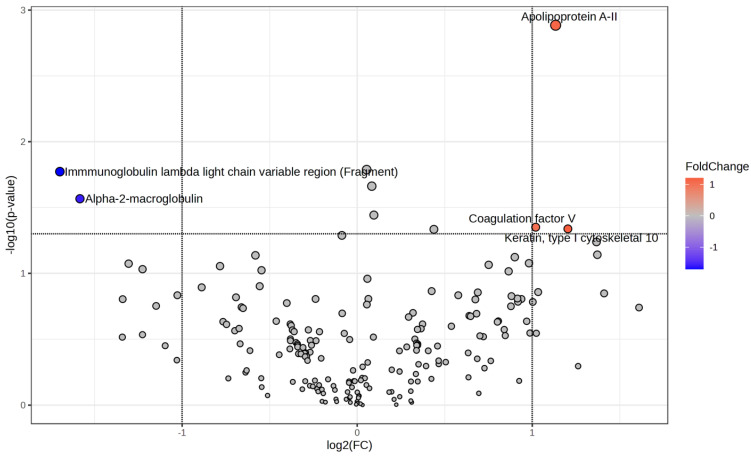
Volcano plot of the serum proteins showing significant variation (FC threshold = 2; *p* value < 0.05) in horses with equine gastric ulcer syndrome after treatment.

**Figure 4:**
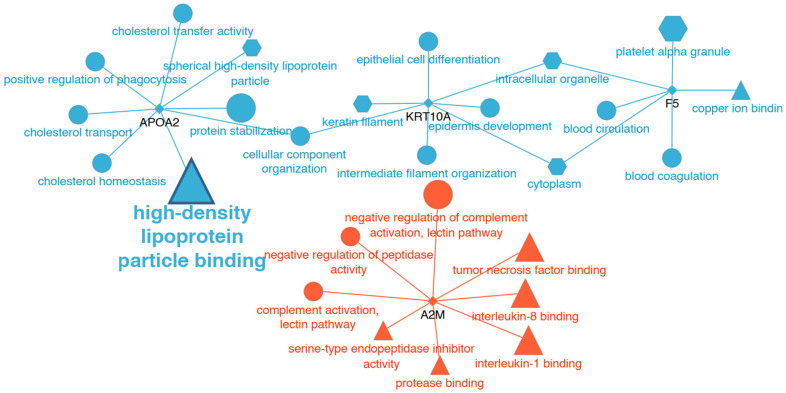
Network-like visualisation of significantly enriched GO annotations of the proteins found differentially expressed in serum. Small, diamond-shaped nodes with black labels represent proteins. Edges link proteins with their corresponding, significantly enriched GO terms. GO terms are represented with shapes according to the ontology they belong to (‘cellular component’ (hexagons), ‘biological process’ (circles), and ‘molecular function’ (triangles)) and with a size that is proportional to their significance (the lower the *p*-value, the larger the node size). Over-expressed proteins and their GO terms are in blue, under-expressed proteins and terms are in red, and grey indicates that a term is equally shared between over- and under-expressed proteins. Only the highlighted GO-term (‘high-density lipoprotein particle binding’) was found to be significantly enriched (*p* < 0.05).

After proteomic analysis, 206 proteins were observed in the serum samples. Three showed higher abundances (keratin, apolipoprotein A-II [Apo-A II], and coagulation factor V) and two lower abundances (immunoglobulin lambda and alpha-2-macroglobulin) in horses after treatment (Table 2) (Figure 3). After GO analysis, the serum proteins were associated with different GO terms, but only the high-density lipoprotein particle binding GO term was found to be significantly enriched (Figure 4, Supplementary Table S2).

## 4. Discussion

In this report, using TMT labelling and liquid proteomics, 13 proteins were identified to be changed in saliva and 5 in the serum of horses between the time of EGUS diagnosis and after a successful treatment verified by gastroscopy. In a previous study using 2D-gel proteomics, only one protein, thioredoxin, was found to change, showing a decrease, in the saliva of horses that recovered from EGUS [12]. Liquid proteomics allowed an expansion of the number of proteins identified, indicating a higher sensitivity of this technique compared to 2D-gel [29].

In this study, the differences were assessed by combining the *t*-test (raw *p*-value), FC threshold, and FDR values. We will focus the discussion on the proteins showing raw *p*-values lower than *p* < 0.05, although only two of them in the case of saliva (UPAR/Ly6 domain-containing protein and peroxiredoxin-1) and one of them in the case of serum (apolipoprotein A-II) also showed an FDR lower than 0.05. This approach was taken to ensure a comprehensive representation of the implicated proteins and their metabolic routes. However, we acknowledge that this decision introduces a limitation to our study, as it may affect the robustness of our findings and the potential for false positives, and therefore the results of this report should be taken with caution.

The analysis of saliva revealed a different set of proteins that changed post-treatment compared to serum. Only one of the proteins that showed significant differences after treatment in saliva was also identified in serum, but it did not show differences in this fluid (Ig-like domain-containing protein). For serum, three proteins (keratin type I cytoskeletal 10, alpha-2-macroglobulin, and immunoglobulin lambda light chain variable region) that showed significant changes were also detected in saliva, but they did not show significant changes in this fluid. Overall, saliva exhibited a greater number of proteins with significant variations compared to serum. These data agree with a previous study on proteomics in horses with EGUS at diagnosis, where there was no substantial match between the patterns of proteome changes in saliva and serum [10]. In addition, in this previous report, horses with EGUS, when compared to healthy horses, showed more proteins with significant variations in saliva than in serum. Also, these variations mirrored many physiological changes and altered mechanisms. Similar results have been described in proteomic studies of different diseases in other species, such as canine mammary tumours [30] and in cows with mastitis [31]. These data support the different changes in the composition of saliva and serum in states of disease and indicate that both fluids can provide complementary information.

The protein that showed a higher increase in saliva after successful treatment in horses was vimentin. This protein has been described to be involved in wound healing and also in the suppression of inflammation, especially in the gastrointestinal tract, where it plays a crucial role in protection from colitis induction [32]. Vimentin coordinates balanced signals, by regulating fibroblast proliferation and epithelial-to-mesenchymal transition, two significant cellular mechanisms that occur in wound repair. The absence of vimentin inhibits these cellular processes, causing delayed wound re-epithelialization and chronic inflammation in injured lesions [33]. In addition, a loss of vimentin leads to delayed wound healing due to impaired directional migration and the contraction of mesenchymal repair cells [34]. Therefore, the increases in vimentin found in our report could be related to the ulcers healing, as induced by the treatment of EGUS. However, it is also important to consider that omeprazole, especially at high doses, has been shown to possess anti-inflammatory properties by decreasing the release of pro-inflammatory cytokines such as TNF-α and IL-1β. Thus, further experiments are needed to fully differentiate whether the reduction in inflammation indicated by vimentin was solely due to the healing of gastric ulcers, or also partially due to the anti-inflammatory effects of omeprazole. UPAR/Ly6 was the only protein that showed significant increases in saliva in terms of both raw *p* values and FDR, being related to the function of the immune system and epithelium, [35] and in addition, has been described to be involved in wound healing repair [36].

Podocalyxin, on the other hand, showed the most significant decrease in saliva after successful EGUS treatment. Podocalyxin is a glycosylated cell surface sialomucin of the CD34 family. A well-established mechanism of action for this protein is through the induction of the metalloprotease (MMP) family. MMPs are a family of proteolytic enzymes that degrade the extracellular matrix (ECM) [37]. Therefore, high values of podocalyxin could be related to an increase in proteolysis and cellular damage. In addition, high values have been associated with a worse prognosis and condition in different neoplasms [38]. In this line, podocalyxin is a prognostic biomarker for poor survival in gastric and oesophageal adenocarcinoma treated with surgery up-front [38]. In this context, the overexpression of this protein might adversely affect ulcer healing. The low podocalyxin level detected in saliva in our study could indicate that podocalyxin was upregulated prior to treatment due to ongoing inflammation/cellular damage, followed by an improvement in these conditions. Another protein that was reduced after treatment in our study was peroxiredoxin I, which showed significant changes in terms of raw *p* values and FDR. This protein’s expression was significantly elevated in rats with ulcerative colitis, and silencing peroxiredoxin I expression improved colon injury by reducing inflammatory responses and intestinal epithelial cell apoptosis [39].

The protein that showed the highest fold change in serum with successful EGUS treatment was keratin type I, that increases after treatment. Keratin 1 and its heterodimer partner keratin 10 are major constituents of the intermediate filament cytoskeleton, contributing to the integrity of the epithelium. Therefore, a decrease in or absence of this protein is associated with inflammation and disease in epithelial tissues [40]. In a previous report from our laboratory, keratin type II was found to be lower in the saliva of horses with EGUS compared to healthy horses [12]. Overall, this could indicate that an increase in keratin type I would contribute to the reparation of the gastric ulcer and the integrity of the squamous epithelium. Apo-A II was the only protein that showed significant increases in serum in terms of both raw *p* values and FDR after successful EGUS treatment, being related to the increase in the expression of the metabolic pathways associated with high-density lipoprotein particle binding, which was found as a GO term in our study. The increase in other apolipoproteins has previously been related to reduced gastric secretion and the healing of gastric ulcers in mice [41].

After treatment, we observed a decrease in serum immunoglobulin lambda. Previous studies [12] reported an increase in various immunoglobulin types, such as immunoglobulin-like domains and immunoglobulin-heavy constant mu, in the saliva of horses with EGUS compared to controls. The decrease in immunoglobulins in horses with EGUS after the treatment found in our study may suggest a reduction in the immune response, that is described to be activated in EGUS pathogenesis [10]. These findings align with the results of the GO metabolic pathways in our report, which indicated a decrease in the immune response after treatment.

This study has various limitations. One is the lack of a control group of horses without EGUS receiving omeprazole, which could be used to elucidate whether omeprazole caused any modifications in protein regulation. In addition, the proteins that showed significant changes should be validated in a larger number of horses to demonstrate their use in monitoring the treatment of EGUS. For the large-scale validation of these results, ideally, high-throughput assays for the quantitation of the main proteins that had different abundance levels between the groups should be developed. Also, it would be of interest to explore if alterations to the horses’ feeding schedules, exercise routines, or other management practices as a part of EGUS treatment could have significant effects on stress levels and immune responses, with changes in proteins in the saliva related to these processes. In addition, in this report, only horses with both ESGD and EGGD were included, and further studies should be performed in order to elucidate if EGGD and ESGD would display different specific biomarkers during treatment progress.

## 5. Conclusions

As a conclusion of this report, it can be stated that 13 proteins in saliva and 5 in the serum samples showed significant changes in horses diagnosed with both ESGD and EGGD between the time of diagnosis and after a successful treatment period. Certain proteins that were found to increase in saliva, such as vimentin, are involved in wound healing, while others that were found to decrease with successful treatments, such as podocalyxin and peroxiredoxin I, are linked to proteolysis and inflammation. In serum, an increase in proteins like keratin type I could be related to the reparation of the gastric ulcer and epithelial integrity, and a reduction in proteins such as immunoglobulin lambda could indicate a reduced immune response. These findings create new avenues for discovering potential biomarkers to monitor EGUS treatment, which is crucial for managing this prevalent disease.

## Figures and Tables

**Table 1:** Changes in the salivary proteome of horses with equine gastric ulcer syndrome (EGUS) after treatment.

Accession	Protein Name	FC *	log2(FC)	*p* Value **	FDR	Regulation after Treatment
F7B5C4	Vimentin	3.32	5.05	0.032	0.08	Up
F6W179	UPAR/Ly6 domain-containing protein	6.08	2.61	0.017	0.03	Up
A0A3Q2I444	Lipocalin/cytosolic fatty-acid-binding domain-containing protein	4.40	2.14	0.037	0.08	Up
A0A3Q2GTN6	Heat shock protein beta-1	3.92	1.97	0.027	0.05	Up
A0A3Q2I2H1	Ig-like domain-containing protein	3.41	1.77	0.040	0.06	Up
A0A9L0T6K3	Peroxiredoxin-5	3.24	1.70	0.043	0.12	Up
A0A5F5PGR5	Chromosome 31 C6orf118 homolog	2.72	1.44	0.039	0.09	Up
F6VRL6	Palmitoyltransferase	0.48	−1.05	0.028	0.06	Down
F6ZRN7	Peptidase inhibitor 3	0.39	−1.36	0.017	0.05	Down
A0A3Q2I4T2	Alpha-1,4 glucan phosphorylase	0.33	−1.60	0.048	0.07	Down
A0A5F5Q0T2	Thioredoxin	0.30	−1.74	0.041	0.06	Down
F6S6J4	Peroxiredoxin-1	0.23	−2.09	0.014	0.03	Down
A0A5F5PWL5	Podocalyxin	0.15	−2.70	0.043	0.05	Down

* FC was obtained as the mean abundance of the after-treatment abundance divided by the mean of before-treatment abundance. ** Obtained by a paired *t*-test. FDR: false discovery rate.

**Table 2:** Changes in the serum proteome of horses with equine gastric ulcer syndrome (EGUS) after treatment.

Accession	Protein Name	FC *	log2(FC)	*p* Value **	FDR	Regulation after Treatment
A0A9L0R3G9	Keratin, type I cytoskeletal 10	2.30	1.20	0.046	0.13	Up
A0A9L0R4P3	Apolipoprotein A-II	2.19	1.13	0.001	0.03	Up
F7DZ01	Coagulation factor V	2.03	1.02	0.045	0.09	Up
F6RI47	Alpha-2-macroglobulin	0.33	−1.58	0.027	0.06	Down
A0A0A1E4B1	Immunoglobulin lambda light chain variable region (Fragment)	0.31	−1.70	0.017	0.05	Down

* FC was obtained as the mean abundance of the after-treatment abundance divided by the mean of before-treatment abundance. ** Obtained by a paired *t*-test. FDR: false discovery rate.

## Data Availability

The raw data supporting the conclusions of this article will be made available by the authors on request.

## Supplementary Materials

The following supporting information can be downloaded at: https://www.mdpi.com/article/10.3390/ani14213105/s1, Table S1: Salivary enriched Gene Ontology pathways associated with differentially expressed proteins in horses with equine gastric ulcer syndrome following successful treatment; Table S2: Serum enriched Gene Ontology pathways associated with differentially expressed proteins in horses with equine gastric ulcer syndrome following successful treatment.
